# Suppression of Ku70/80 or Lig4 leads to decreased stable transformation and enhanced homologous recombination in rice

**DOI:** 10.1111/j.1469-8137.2012.04350.x

**Published:** 2012-10-10

**Authors:** Ayako Nishizawa-Yokoi, Satoko Nonaka, Hiroaki Saika, Yong-Ik Kwon, Keishi Osakabe, Seiichi Toki

**Affiliations:** 1 Plant Genome Engineering Research Unit, National Institute of Agrobiological Sciences2-1-2 Kannondai, Tsukuba, Ibaraki 305-8602, Japan; 2 Graduate School of Life and Environmental Sciences, University of Tsukuba1-1-1 Tennodai, Tsukuba, Ibaraki 305-8577, Japan; 3 Kihara Institute for Biological Research, Yokohama City University641-12 Maioka-cho, Yokohama 244-0813, Japan; 4 Institute for Environmental Science and Technology, Saitama UniversityShimo-Okubo 255, Sakura-ku, Saitama-shi, 338-8570 Japan

**Keywords:** DNA double-strand breaks (DSBs), *DNA ligase 4* (*Lig4*), homologous recombination (HR), *Ku70*, *Ku80*, nonhomologous end joining (NHEJ), *Oryza sativa* (rice), transferred DNA (T-DNA)

## Abstract

- Evidence for the involvement of the nonhomologous end joining (NHEJ) pathway in *Agrobacterium*-mediated transferred DNA (T-DNA) integration into the genome of the model plant Arabidopsis remains inconclusive.
- Having established a rapid and highly efficient *Agrobacterium*-mediated transformation system in rice (*Oryza sativa*) using scutellum-derived calli, we examined here the involvement of the NHEJ pathway in *Agrobacterium*-mediated stable transformation in rice. Rice calli from *OsKu70*,*OsKu80* and *OsLig4* knockdown (KD) plants were infected with *Agrobacterium* harboring a sensitive emerald luciferase (*LUC*) reporter construct to evaluate stable expression and a green fluorescent protein (GFP) construct to monitor transient expression of T-DNA.
- Transient expression was not suppressed, but stable expression was reduced significantly, in KD plants. Furthermore, *KD-Ku70* and *KD-Lig4* calli exhibited an increase in the frequency of homologous recombination (HR) compared with control calli. In addition, suppression of *OsKu70*,*OsKu80* and *OsLig4* induced the expression of HR-related genes on treatment with DNA-damaging agents.
- Our findings suggest strongly that NHEJ is involved in *Agrobacterium*-mediated stable transformation in rice, and that there is a competitive and complementary relationship between the NHEJ and HR pathways for DNA double-strand break repair in rice.

## Introduction

Plant cells are continually exposed to endogenous and exogenous genotoxic stresses, such as reactive oxygen species and UV light, which lead to the accumulation of numerous types of DNA damage, including cross-linking of DNA, base oxidation or alkylation, mismatch of bases, DNA single-strand breaks and DNA double-strand breaks (DSBs). DSBs are amongst the most serious types of DNA damage in living cells and can lead to cell death if not repaired. There are at least two repair pathways for DSB repair: nonhomologous end joining (NHEJ), which involves rejoining of the broken DNA ends; and homologous recombination (HR), which is an accurate pathway that uses homologous DNA sequences from the sister chromatid as a template. NHEJ is used preferentially to deal with DSBs in higher eukaryotes, including higher plants (Mladenov & Iliakis, 2011; Symington & Gautier, 2011; Waterworth *et al*., 2011), whereas HR is the main DSB repair pathway in bacteria and yeast (Pâques & Haber, 1999; Aylon & Kupiec, 2004).

The NHEJ pathway in vertebrates is thought to be as follows. A Ku70/Ku80 complex binds initially to two DNA ends at the DSB site, and then recruits a DNA-dependent protein kinase (DNA-PKcs), which has not been identified in plants. DNA-PKcs phosphorylates and activates many proteins, including nuclease and itself. Ultimately, the Lig4–Xrcc4 complex rejoins the two DNA ends of the break (Mladenov & Iliakis, 2011; Symington & Gautier, 2011). Many proteins involved in NHEJ found in mammalian cells have also been identified in Arabidopsis and rice (reviewed by Singh *et al*., 2010; Edlinger & Schlögelhofer, 2011), including Arabidopsis Ku70 (Tamura *et al*., 2002), rice Ku70 (Hong *et al*., 2010), Arabidopsis Ku80 (Tamura *et al*., 2002), Arabidopsis DNA ligase 4 (Lig4) (West *et al*., 2000) and Arabidopsis Xrcc4 (West *et al*., 2000).

In Arabidopsis, mutants of the *ku70*, *ku80* and *lig4* genes have been shown to display hypersensitivity to DSB-inducing agents, including γ-irradiation, methyl methanesulfonate, ionizing radiation and bleomycin (van Attikum *et al*., 2003; Friesner & Britt, 2003; Gallego *et al*., 2003; Li *et al*., 2005; Hong *et al*., 2010; Wang *et al*., 2010), indicating that Ku70/Ku80 and Lig4 proteins play an important role in DSB repair in Arabidopsis. In addition, recent studies in mammalian cells have shown that DSBs can be rejoined in the presence of chemical inhibition or mutation of key NHEJ factors, such as DNA-PKcs, Ku70, Ku80, Lig4 and XRCC4, suggesting the existence of backup pathways for NHEJ (Mladenov & Iliakis, 2011; Symington & Gautier, 2011). Such a backup NHEJ pathway may utilize poly(ADP-ribose) polymerase-1, MRN, histone H, DNA ligase III and XRCC1 (Cheng *et al*., 2011; Mladenov & Iliakis, 2011; Symington & Gautier, 2011). In Arabidopsis, several studies have also provided evidence for the existence of Ku- and Lig4-independent pathways, and have identified proteins, such as AtLig1 and AtXRCC1, that function in these pathways (Charbonnel *et al*., 2010, 2011; Waterworth *et al*., 2009, 2011).

*Agrobacterium*-mediated plant genetic transformation uses the transferred DNA (T-DNA) region on a binary plasmid harbored by *Agrobacterium tumefaciens* as a vector for genetic engineering. DNA repair pathways have been thought to be involved in the integration of T-DNA into the plant genome, and two major models for this integration have been proposed. The first – the DSB repair model – hypothesizes that single-strand T-DNAs imported into the plant cell nucleus by the virulence protein complex are replicated to a double-stranded form and are subsequently integrated into DSBs in the host genome (Gelvin, 2010; Pitzschke & Hirt, 2010; Magori & Citovsky, 2011). By contrast, the second – the strand-invasion model – assumes that the 3′ end of single-stranded T-DNA finds a microhomology to plant DNA and invades the target site host DNA. The VirD2-attached 5′ end of the T-DNA binds to a nick in the plant DNA and is ligated. The complementary strand of the T-DNA is synthesized, resulting in integration of a double-strand copy of the T-DNA into the plant genome (Gelvin, 2010; Pitzschke & Hirt, 2010; Magori & Citovsky, 2011).

In Arabidopsis, mutation of either the *AtKu80* or *AtLig4* gene caused a decrease in the frequency of stable T-DNA integration following an *in planta* floral dip transformation assay (Friesner & Britt, 2003). Involvement of *AtKu80* in T-DNA integration was also observed in a root tumorigenesis assay (Li *et al*., 2005). By contrast, no decrease in T-DNA integration was observed in *AtKu80* mutants using the *in planta* floral dip transformation assay (Gallego *et al*., 2003). Furthermore, the *AtLig4* mutant was not impaired in T-DNA integration using either the *in planta* floral dip or tumorigenesis assay (van Attikum *et al*., 2003). The discrepancy between these previous reports might be attributable to differences in the transformation mechanisms between germline and somatic cells, and could also depend on the physiological condition of the plant material used for the experiments. Thus, evidence for the involvement of the NHEJ pathway in *Agrobacterium*-mediated T-DNA integration into the plant genome remains inconclusive.

The choice of NHEJ and HR pathways for DSB repair in eukaryotes depends on the cell type, cell cycle stage and complexity of the DNA end (Shrivastav *et al*., 2008; Heyer *et al*., 2010; Symington & Gautier, 2011). In addition, disruption of the NHEJ pathway has been shown to lead to an increased frequency of HR in fungi (Ninomiya *et al*., 2004; Villalba *et al*., 2008), mammals (Liang *et al*., 1996; Pierce *et al*., 2001; Allen *et al*., 2003) and plants (Gallego *et al*., 2003), suggesting that the NHEJ pathway competes with the HR pathway for DSB repair. However, the *AtKu80* mutant did not show an enhanced frequency of intrachromosomal HR (Gallego *et al*., 2003).

We have developed a stable and efficient *Agrobacterium*-mediated transformation system for rice (Toki *et al*., 2006). Most recently, we have further constructed a sequential monitoring system for stable transformation by visualizing cells in which T-DNA is successfully integrated into the rice genome using a nondestructive and highly sensitive visible marker in rice (H. Saika *et al*., unpublished). In this report, we investigated the involvement of the NHEJ pathway in an *Agrobacterium*-mediated stable transformation system, and the choice between NHEJ and HR for DSB repair in rice plants. The evidence presented here indicates that the NHEJ pathway participates in *Agrobacterium*-mediated stable transformation in rice, and that this pathway possibly competes with the HR pathway for DSB repair. Alternatively, an increase in the HR pathway might result from genome instability and the up-regulation of HR genes derived from the suppression of the NHEJ pathway in rice somatic cells.

## Materials and Methods

### Plant materials

*Oryza sativa* L. cv Nipponbare (genetic background of *KD-Ku70*, *KD-Ku80* and *KD-Lig4*) and *O. sativa* cv Dongjin (genetic background of *OsKu70* T-DNA insertional line) were used in this study. The *OsKu70* T-DNA insertional line was obtained from the Rice T-DNA Insertion Sequence Database (http://signal.salk.edu/cgi-bin/RiceGE). Plant genotypes were determined by PCR using the T-DNA right border primer pGA2715 RB (5′-ttggggtttctacaggacgtaac-3′) and *OsKu70* gene-specific primers (5′-ccaaccttagtttcactcttgttacgtg-3′ and 5′-ggaaagcctaagtgacatcactggaa-3′).

### Generation of transgenic plants

Vectors for the generation of *OsKu70-*, *OsKu80-* and *OsLig4*-suppressed rice plants using the RNA interference (RNAi) method were constructed with the vector pANDA (Miki & Shimamoto, 2004). The 3′ end of *OsKu70*, *OsKu80* or *OsLig4* cDNA as an RNAi trigger was amplified from first-strand cDNA by PCR using the following primer sets, *OsKu70* RNAi (forward 5′-cacccggtggtggacttgaaatct-3′ and reverse 5′-ctctgcagactggagtgacatt-3′), *OsKu80* RNAi (forward 5′-cacccttctgtctgaaacccgagc-3′ and reverse 5′-cagagcttctggaggtgagg-3′), *OsLig4* RNAi (forward 5′-caccacaccgctgaaacaacgagta-3′ and reverse 5′-ggcgacgtccttgtaactgac-3′), and was cloned into the vector pENTR/D-TOPO using directional TOPO cloning methods (Life Technologies, Carlsbad, CA, USA) to yield an entry vector. The RNAi trigger fragments of *OsKu70* (325 bp), *OsKu80* (339 bp) and *OsLig4* (341 bp) were re-cloned into the RNA silencing binary pANDA vector using a Gateway LR clonase reaction (Life Technologies).

The LU-UC recombination substrate was constructed as follows. The 120-bp artificial synthesized fragment containing a multi-cloning site and two I-*Sce*I recognition sites (*Asc*I-*Sac*I-I-*Sce*I-*Avr*II-*Aat*II-I-*Sce*I-*Csp*45I-*Pac*I) was cloned into the *Asc*I/*Pac*I site of pZAmI, which is a derivative of pPZP201 (Hajdukiewicz *et al*., 1994), with a herbicide bispyribac sodium resistance cassette (rice acetolactate synthase (ALS) promoter 3 kb + mutant ALS gene (W548L/S627I) (Shimizu *et al*., 2005) + ALS terminator 0.7 kb). The full length and 5′ region of the luciferase (LUC) cDNA were amplified by PCR using pSP-luc+NF vector (Promega) as a template and the following primer pairs: full length (LUC-F 5′-*tctaga*atggtcaccgacgccaaaaacat-3′ (*Xba*I site in italics) and LUC-R 5′-*gagctc*ttacacggcgatctttccgcc-3′ (*Sac*I site in italics)) and 5′ region (LUC-F and LUC-R 891 5′-*gagctc*ggcgaagaaggagaatagggtt-3′ (*Sac*I site in italics)). The full length and 5′ region of LUC cDNA were cloned into the *Xba*I/*Sac*I site between the maize ubiquitin1 promoter + first intron and the transcription terminator of the Arabidopsis ribulose-bisphosphate carboxylase small subunit (rbcS) gene in pENTR (Life Technologies), yielding the vectors pPubi:LUC and pPubi:LU, respectively. The fragment containing the maize ubiquitin1 promoter + first intron and 5′ region of LUC cDNA in the pPubi:LUC vector was digested with *Asc*I/*Sac*I and cloned into pZAmI, yielding pZAmLU. A 3.2-kb fragment containing the promoter sequence of rice elongation factor 1, mutant codA gene (D314A) (Mahan *et al*., 2004) and transcription terminator of the rice glycerol 3-phosphate dehydrogenase gene was amplified from pE_Pef:codAm:Tg3p vector (K. Osakabe *et al*., unpublished) using primers 5′-*cctagg*aagctttataatcccgtgcg-3′ (*Avr*II site in italics) and 5′-*gacgtc*gaattccaagcaccaccgcga-3′ (*Aat*II site in italics). The amplified fragment was integrated into the *Avr*II/*Aat*II site of pZAmLU, yielding pZAmLUcodA. A fragment containing the 3′ region of LUC cDNA (1485 bp) and the transcription terminator of the rbcS gene in pPubi:LUC was digested with *Csp*45I (internal restriction site)/*Pac*I and integrated into pZAmLUcodA, yielding pZAmLUcodAUC. For construction of the I-*Sce*I expression plasmid, the yeast I-*Sce*I gene was cloned into the *Spe*I/*Sac*I site between the 2 × CaMV35 promoter + Ω and the transcription terminator of rice heat shock protein 17.3 of pZDB (K. Osakabe *et al*., unpublished).

These plant binary vectors were transferred into *Agrobacterium tumefaciens* strain EHA105 (Hood *et al*., 1993) by electroporation. *Agrobacterium*-mediated transformation of rice (*O. sativa* cv Nipponbare) was performed as described previously (Toki, 1997; Toki *et al*., 2006).

### RNA extraction and quantitative reverse transcriptase-polymerase chain reaction (RT-PCR) analysis

Total RNA was extracted from calli and seedlings of rice plants using an RNeasy Plant Mini Kit (Qiagen, Valencia, CA, USA). Quantitative RT-PCR was performed with a Power SYBR Green PCR Master Mix (Life Technologies) and an ABI7300 (Life Technologies) according to the manufacturer's protocols. Primer pairs for quantitative RT-PCR were designed using Primer3Plus (http://www.bioinformatics.nl/cgi-bin/primer3plus/primer3plus.cgi) and are as follows: OsAct1 (5′-cattgctgacaggatgagcaa-3′ and 5′-gggcgaccaccttgatctt-3′); OsKu70 (5′-acgtgcaagagatgcacaag-3′ and 5′-aactcctcatcgggcctact-3′); OsKu70 3′ (5′-cggtggtggacttgaaatct-3′ and 5′-gctgacgagtgcctctttct-3′); OsKu80 (5′-agctcaacgtgggttcagac-3′ and 5′-ctccagtgccttttggtgat-3′); OsLig4 (5′-ttggtgaatgcggactacaa-3′ and 5′-aatgctgcacacttgaccac-3′); OsRad51A2 (5′-tggtggacgcttggattgat-3′ and 5′-caggattccagggcgctat-3′); OsBRCA1 (5′-tgcaatctgcaacctctttg-3′ and 5′-agccctgtgcatcttagatttc-3′); OsPARP2A (5′-gcggtacgttctccatgttt-3′ and 5′-tacggtttcatctccgtgct-3′).

### *Agrobacterium*-mediated stable transformation assay

The *Agrobacterium*-mediated stable transformation assay was performed according to the method established by H. Saika *et al*. (unpublished). The reporter constructs used (Fig. 2a, p35Smini:Eluc vector) contain the *gfbsd2* gene (Ochiai-Fukuda *et al*., 2006) encoding a fusion gene of *gfp* and *bsd* (herbicide Blasticidin S resistance) under the control of the constitutive rice elongation factor 1 promoter (Pef) and emerald luciferase (Eluc) gene under the control of the CaMV35S minimal promoter (m), which can express the Eluc gene only after stable integration into an enhancer-like sequence (H. Saika *et al*., unpublished). Rice calli from control plants transformed with pANDA empty vector and *KD-OsKu70*, *KD-OsKu80* and *KD-OsLig4* plants were induced and grown on N6D medium containing 50 mg l^−1^ hygromycin for 7 d at 33°C, and were infected with *Agrobacterium* harboring p35Smini:Eluc vector. After 3 d of co-cultivation with *Agrobacterium* at 22–25°C under continuous darkness on 2N6-AS medium, calli were washed and cultured on N6D medium containing 50 mg l^−1^ hygromycin (Wako Pure Chemical Industries, Osaka, Japan), 10 mg l^−1^ blasticidin S (Wako Pure Chemical Industries) and 25 mg l^−1^ meropenem (Wako Pure Chemical Industries). Green fluorescent protein (GFP) fluorescence and LUC luminescence were then detected and quantified sequentially in rice callus transformed with the p35Smini:Eluc reporter construct.

### Observation of GFP fluorescence

GFP fluorescence images were taken using a Molecular Imager FX (Bio-Rad, USA) with an excitation wavelength of 488 nm and an emission wavelength of 530 nm.

### Observation of LUC luminescence

Rice calli were treated with 0.2 mM Beetle Luciferin potassium salt (Promega). LUC luminescence images were taken using a high-resolution photon counting camera (C2400–700 VIM camera, Hamamatsu Photonics, Hamamatsu, Japan) with a 15 min exposure time, and processed using Aquacosmos (Hamamatsu Photonics).

### Genomic DNA extraction and Southern blot analysis

Genomic DNA was extracted from rice calli transformed with the LU-UC recombination substrate using Nucleon PhytoPure (GE Healthcare, Little Chalfont, Buckingham, UK) according to the manufacturer's protocol, digested with *Hin*dIII and fractionated in a 1.0% agarose gel. Southern blot analysis was performed according to a standard protocol. Specific DNA probes for the *codA* gene were synthesized with a PCR digoxigenin (DIG) probe synthesis kit (Roche Diagnostics) according to the manufacturer's protocol, using the primers 5′-ggcccatggtgtcgaataacgctttacaaac-3′ and 5′-cccgagctctcaacgtttgtaatcgatggct-3′.

### Genotoxic stress treatment

Seedlings of control, *KD-Ku70*, *KD-Ku80* and *KD-Lig4* plants were grown on Murashige and Skoog medium for 7 d at 30°C under continuous light in a growth chamber, transferred to water containing 5 μM bleomycin and incubated for 12 h in the growth chamber. The aerial parts of plants were collected and immediately frozen in liquid N_2_ and stored at −80°C for quantitative RT-PCR analysis and microarray analysis.

### Microarray analysis

Total RNA was isolated using an RNeasy Plant Mini Kit (Qiagen), labeled using a Quick-Amp Labeling Kit (Agilent Technologies, Santa Clara, CA, USA) and hybridized to a rice 4 × 44 K custom oligoDNA microarray (Agilent Technologies) according to the manufacturer's instructions. Hybridization microarray slides were scanned with an Agilent Microarray Scanner (Agilent Technologies). The images generated were analyzed using Feature Extraction software (Agilent Technologies), applying standard normalization procedures.

## Results

### Suppression of NHEJ-related gene expression causes inhibition of *Agrobacterium*-mediated stable transformation in rice calli

We searched for genes with high sequence homology to *AtKu80* and *AtLig4* in the rice genome annotation project database (http://rice.plantbiology.msu.edu/), and identified the rice homologs of *Ku80* (LOC_Os03g63920.1) and *Lig4* (LOC_Os04g51700.1). We generated rice plants transformed with RNAi constructs using the 3′ untranslated region (UTR) of *OsKu70*, *OsKu80* or *OsLig4* mRNA. Among a number of T_2_ lines generated, two independent lines of each transgenic rice plant with a single T-DNA insertion and reduced expression of the targeted gene were selected and named *KD-OsKu70* (-1 and -4), *KD-OsKu80* (-1 and -4) and *KD-OsLig4* (-8 and -14), respectively (Fig.a–c). T_0_ plants were self-pollinated and T_1_ seeds were used for further analysis.

**Fig 1:**
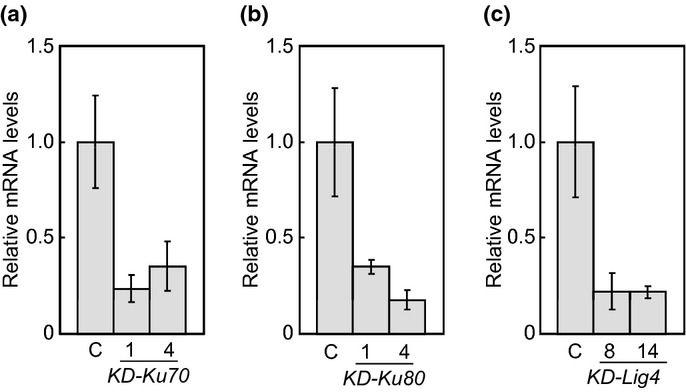
Expression of *OsKu70*, *OsKu80* and *OsLig4* in control, *KD-Ku70*, *KD-Ku80* and *KD-Lig4* transgenic rice (*Oryza sativa*) calli. Total RNA was extracted from 1-wk-old control, *KD-Ku70*, *KD-Ku80* and *KD-Lig4* transgenic rice calli. A quantitative PCR analysis was carried out to determine the expression levels of *OsKu70* (a), *OsKu80* (b) and *OsLig4* (c). Relative transcript levels were normalized to *OsAct1* mRNA. Error bars represent ± SD of three individual experiments.

There were no significant differences between wild-type and *KD-OsKu70*, *KD-OsKu80* and *KD-OsLig4* plants in the frequency of callus induction, callus proliferation, callus shape, vegetative growth or fertility (data not shown).

In previous reports of *Agrobacterium*-mediated transformation in rice calli, two patterns of expression derived from the foreign gene on the T-DNA have been detected: transient expression of the foreign gene from the unintegrated double-stranded T-DNA, and stable expression following integration of the foreign gene into the plant genome (Toki *et al*., 2006). To distinguish between transient and stable expression more clearly and sensitively, we constructed a new T-DNA vector, p35Smini:Eluc, containing the *GFP* gene under the control of a constitutive promoter and the emerald *LUC* gene under the control of a minimal promoter (Fig.a; H. Saika *et al*., unpublished). This system allows us to evaluate *Agrobacterium*-mediated transformation events by visualizing stable transgene expression in rice. The GFP signal observed in rice calli at the early transformation stage (3–4 d after *Agrobacterium* infection) was derived from transient expression of T-DNA that was not integrated into the rice genome. The emerald LUC luminescence seen in rice calli at later transformation stages (7–9 d after *Agrobacterium* infection) was derived from stable expression of T-DNA that had integrated successfully into the rice genome and was expressed because of a nearby enhancer.

**Fig 2:**
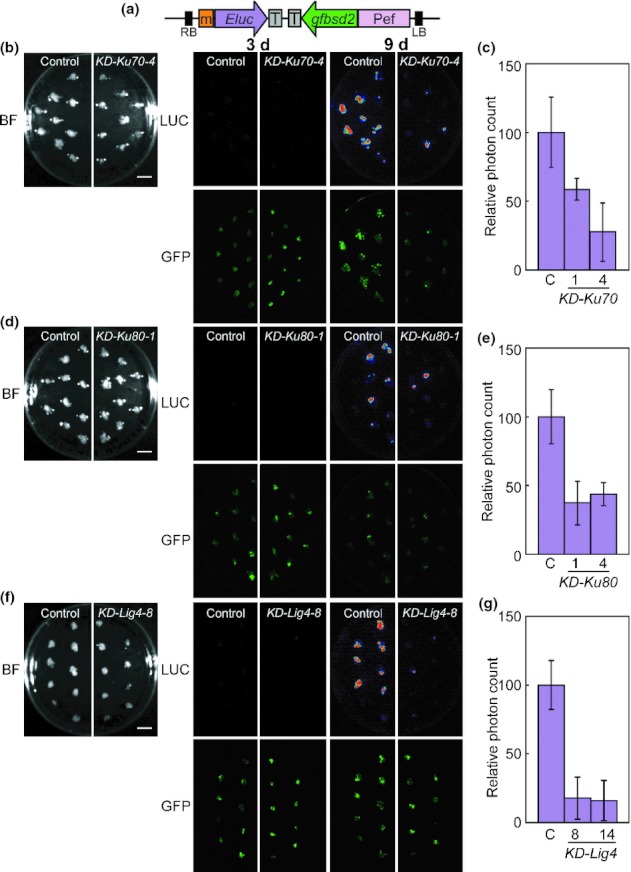
Efficiency of stable transformation in control and *KD-OsKu70* (b, c), *KD-OsKu80* (d, e) and *KD-OsLig4* (f, g) transgenic rice (*Oryza sativa*) calli. (a) Schematic diagram of the reporter constructs (p35Smini:Eluc) for the *Agrobacterium*-mediated stable transformation assay. The reporter constructs (p35Smini:Eluc vector) contain the *gfbsd2* gene (Ochiai-Fukuda *et al*., 2006) encoding a fusion gene of green fluorescent protein (*GFP*) and *bsd* (herbicide Blasticidin S resistance) under the control of the constitutive rice elongation factor 1 promoter (Pef) and emerald luciferase (*LUC*) gene under the control of the CaMV35S minimal promoter (m), which can express the *LUC* gene only after stable integration into an enhancer-like sequence. T, transcription terminator; LB, left border; RB, right border. (b, d, f) Representative bright-field image (BF, left panel), LUC luminescence image (LUC, upper right panel) and GFP fluorescence image (GFP, lower right panel) of control, and *KD-OsKu70* (b), *KD-OsKu80* (d) and *KD-OsLig4* (f) transgenic rice calli. The GFP signal observed on rice calli at an early transformation stage (3 d after *Agrobacterium* infection) was derived from the transient expression of T-DNA which was not integrated into the rice genome. The LUC luminescence, as represented by the false color scale, on rice calli at the late transformation stage (after 9 d of *Agrobacterium* infection) was derived from the stable expression of T-DNA which was then successfully integrated into the rice genome. Bars, 1 cm. (c, e, g) Graphical representation of LUC luminescence intensity on control, and *KD-OsKu70* (c), *KD-OsKu80* (e) and *KD-OsLig4* (g) transgenic rice calli at 9 d after infection. Relative LUC luminescence levels were normalized to LUC luminescence levels of control calli (= 100). *Agrobacterium*-mediated stable transformation assays were performed using three independent batches. Error bars represent ±SD.

To test the efficiency of *Agrobacterium*-mediated stable transformation, calli from control plants transformed with the empty pANDA vector and from *KD-OsKu70*, *KD-OsKu80* and *KD-OsLig4* plants were induced and grown on N6D medium for 7 d. Calli were infected with *Agrobacterium* harboring the p35S mini:Eluc vector. At 3 d after infection, transient expression of GFP derived from nonintegrated T-DNA in *KD-OsKu70*, *KD-OsKu80* and *KD-OsLig4* calli was comparable with that in control calli (Fig.b,d,f), whereas LUC signals, monitoring stable expression of the transgene in *KD-OsKu70*-1, *KD-OsKu70*-4, *KD-OsKu80*-1, *KD-OsKu80*-4, *KD-OsLig4*-8 and *KD-OsLig4*-14 calli, were decreased to 60, 30, 40, 45, 20 and 20% of that in control calli at 9 d after infection (Fig.b–g).

**Fig 3:**
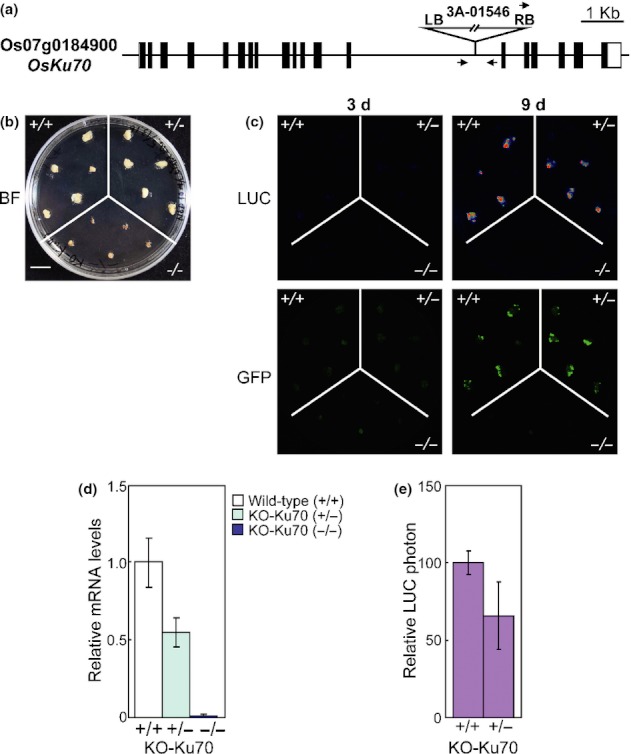
Efficiency of stable transformation in wild-type and *KO-OsKu70* mutant rice (*Oryza sativa*) calli. (a) Schematic diagram of the *OsKu70* (Os07g0184900) gene showing the position of the inserted T-DNA (3A-01546). Open and solid bars show the untranslated regions and coding region, respectively. The primer sets used in genotyping are represented by arrows. (b,c) Bright-field image (BF, left panel), luciferase luminescence image (LUC, upper right panel) and green fluorescent protein fluorescence image (GFP, lower right panel) of wild-type (+/+), *KO-OsKu70* heterozygous mutant (+/−) and *KO-OsKu70* homozygous mutant (−/−) rice calli. Bar, 1 cm. The Green fluorescent protein (GFP) signal observed on rice calli at an early transformation stage (3 d after *Agrobacterium* infection) was derived from the transient expression of T-DNA which was not integrated into the rice genome. Luciferase luminescence (LUC), as represented by the false color scale, on rice calli at the late transformation stage (9 d after *Agrobacterium* infection) was derived from the stable expression of T-DNA which was integrated successfully into the rice genome. Bar, 1 cm. (d) Quantitative reverse transcriptase-polymerase chain reaction (RT-PCR) analysis of *OsKu70* transcripts in wild-type (+/+), *KO-OsKu70* heterozygous mutant (+/−) and *KO-OsKu70* homozygous mutant (−/−)rice calli. Error bars represent ± SD of three individual experiments. (e) Graphical representation of LUC luminescence intensity on wild-type (+/+), *KO-OsKu70* heterozygous mutant (+/−) and *KO-OsKu70* homozygous mutant (−/−) rice calli at 9 d after infection. Relative LUC luminescence levels were normalized to LUC luminescence levels of control calli (= 100). *Agrobacterium*-mediated stable transformation assays were performed using three independent batches. Error bars represent ± SD.

Furthermore, a mutant line (3A-01546; Hong *et al*., 2010; Fig. 3a), containing a T-DNA insertion in the *OsKu70* gene (*KO-Ku70*), was acquired from the Rice T-DNA Insertion Sequence Database (http://www.postech.ac.kr/life/pfg/risd/) and self-fertilized to obtain a pure homozygous line. However, progeny derived from homozygous T-DNA insertion lines for the *OsKu70* gene (*KO-Ku70*^−/−^) were not obtained. It has been reported that plants homozygous for the *OsKu70* T-DNA insertion mutation display a sterile phenotype and severely retarded vegetative organ growth (Hong *et al*., 2010). Thus, mature seeds of the heterozygous *KO-Ku70* mutant (*KO-Ku70*^+/−^) were analyzed for segregation of the mutant allele and transcript levels of the *OsKu70* gene (Fig.d). The seeds were then inoculated on N6D medium to induce calli. As 7-d-old calli of *KO-Ku70*^−/−^ showed severely reduced growth compared with calli of wild-type or knockdown (KD) plants (Fig.b), we could not use these calli for the *Agrobacterium*-mediated stable transformation assay. Seven-day-old rice calli of the control and *KO-Ku70*^+/−^ plants were infected with *Agrobacterium* harboring p35S mini:Eluc vector, and GFP fluorescence and LUC luminescence were analyzed after 3 d of co-culture and elimination of the bacteria. Consistent with results from *KD-Ku70*, no significant differences in the transient expression of GFP between control and *KO-Ku70*^+/−^ plants were observed (Fig.c). However, LUC luminescence was suppressed slightly in *KO-Ku70*^+/−^ calli when compared with control calli at 9 d after infection (Fig.c,e). These results suggest that the suppression of NHEJ-related gene expression causes an inhibition of *Agrobacterium*-mediated stable transformation, but not of import of T-DNA into the nuclei of infected cells.

### Suppression of NHEJ-related gene expression leads to enhanced frequency of HR

To investigate whether suppression of the NHEJ pathway causes an increase in HR frequency, we used an HR assay system that permits recombination events to be visualized as luminescence from a reconstituted recombination substrate locus. This recombination substrate consists of two partially duplicated fragments of the LUC gene (LU-UC) interrupted by a cytosine deaminase (*codA*) expression cassette and two recognition sites for meganuclease, I-*Sce*I (Fig.a). These fragments of the LUC gene share a duplicated 720-bp region in tandem arrangement. We generated rice plants transformed with recombination substrate (LU-UC), and evaluated the number of integration loci of the transgene. Southern blot analysis with a CodA probe showed that almost all transformants contained a single copy or two copies of the T-DNA (data not shown). Transgenic lines with a single copy (LU-UC lines 8) and two copies (LU-UC lines 9) of the LU-UC recombination substrate were selected (Fig.b), and were transformed with *Agrobacterium* to introduce pANDA empty vector (as a control) or the KD constructs of *OsKu70* or *OsLig4* (Fig.c).

**Fig 4:**
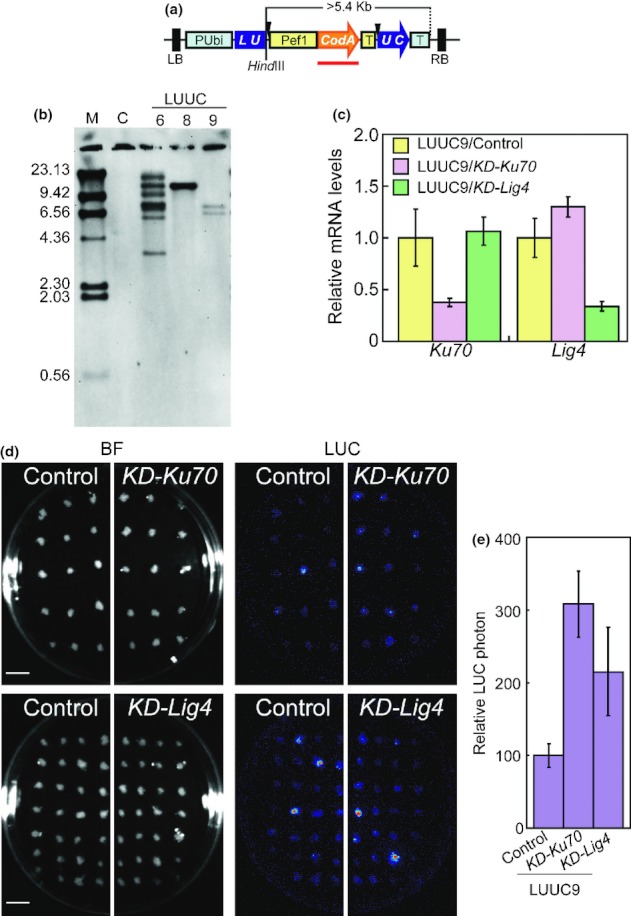
Luciferase (LUC) luminescence of recombination events by spontaneous double-strand breaks (DSBs) in control, *KD-OsKu70* and *KD-OsLig4* rice (*Oryza sativa*) calli. (a) Schematic diagram of the LU-UC recombination substrate. This recombination substrate consists of two partially duplicated fragments of the LUC gene interrupted by a cytosine deaminase (codA) expression cassette and two recognition sites for the meganuclease I-*Sce*I (arrowheads). PUbi, maize ubiquitin1 promoter; Pef1, rice elongation factor 1 promoter; T, transcription terminator; LB, left border; RB, right border. Red bar represents DNA probe for Southern blot analysis. (b) Southern blot analysis using a codA probe (shown by the red bar in a) and 1 μg of genomic DNA extracted from wild-type and LU-UC recombination substrate transgenic rice calli and digested with *Hin*dIII. (c) Quantitative reverse transcriptase-polymerase chain reaction (RT-PCR) analysis of *OsKu70* (left) and *OsLig4* (right) transcripts in LU-UC-9 transgenic lines transformed with pANDA empty vector (as a control), *KD-Ku70* or *KD-Lig4*. Relative transcript levels were normalized to *OsAct1* mRNA. Error bars represent ± SD of three individual experiments. (d) Bright-field image (BF) and LUC luminescence image (LUC) of control, *KD-Ku70* (upper panel) and *KD-Lig4* (lower panel) rice calli. LUC luminescence derived from recombination events of recombination substrate by spontaneous DSB were detected in 4-wk-old transgenic rice calli without induction of I-*Sce*I expression. Bar, 1 cm. (e) Graphical representation of the LUC luminescence intensity on control and *KD-Ku70* and *KD-Lig4* rice calli. Relative LUC luminescence levels were normalized to LUC luminescence levels of control calli (= 100). Error bars represent ± SD.

Mature seeds of T_1_ control, *KD-Ku70* and *KD-Lig4* transgenic (T_2_ LU-UC transgenic) plants were grown and selected on N6D medium containing hygromycin and bispyribac without 5-fluorocytosine, which is converted to toxic 5-fluorouracil by codA. The negative selection by codA results in the preferential growth of calli in which the codA expression cassette has been removed from the recombination substrate locus. After 4 wk of selection, LUC luminescence derived from reconstituted recombination substrate was analyzed on transgenic calli. *KD-Ku70* and *KD-Lig4* transgenic calli containing two copies of the recombination substrate showed a two- to three-fold increase in the frequency of HR compared with control calli (Fig.d,e). However, LUC luminescence was not detected on transgenic calli containing a single copy of LU-UC (data not shown).

Next, we investigated the effect of suppression of NHEJ on the frequency of DSB-inducible HR using transient I-SceI expression. To introduce DSBs at the two I-SceI sites flanking the codA expression cassette of LU-UC recombination substrates by inducing transient I-*Sce*I expression, 4-wk-old control, *KD-OsKu70* and *KD-OsLig4* transgenic calli containing LU-UC recombination substrates were infected with *Agrobacterium* harboring the I-*Sce*I expression vector (Supporting Information Fig. S1a). LUC luminescence derived from DSB-inducible reconstituted recombination substrate was analyzed 5 d after infection, at which point T-DNA is largely unintegrated into the rice genome (Toki *et al*., 2006). We found no difference in LUC luminescence between control, *KD-OsKu70* and *KD-OsLig4* calli (Fig. S1b).

### Effect of suppression of NHEJ-related gene expression on induction of DSB-inducible genes

The enhancement of HR frequency observed in *KD-OsKu70* and *KD-OsLig4* calli could be explained by competition for DNA repair between HR and NHEJ at the sites of DSBs. However, it could also be explained by the induction of HR-related gene expression. We analyzed the effect of suppression of NHEJ-related gene expression on the transcript levels of *OsRad51A2*, which plays a crucial role in HR repair of DSBs (Rajanikant *et al*., 2008), and *OsBRCA1*, which colocalizes with Rad51 at DSB sites and activates HR via Rad51 (Lafarge & Montané, 2003). Seven-day-old control, *KD-OsKu70*, *KD-OsKu80* and *KD-OsLig4* rice plants were either treated with 5 μM bleomycin, which generates DSBs and DNA single-strand breaks, or left untreated for 12 h. No significant differences were observed in the levels of *OsRad51A2* and *OsBRCA1* transcripts between controls and transgenic plants without bleomycin treatment (Fig.a,b), whereas, on treatment with 5 μM bleomycin for 6 and 12 h, transcripts of both genes increased markedly in transgenic relative to control plants (Fig.a,b). Suppression of *OsKu70* expression showed the highest transcript levels of HR-related genes at 12 h after treatment with bleomycin.

**Fig 5:**
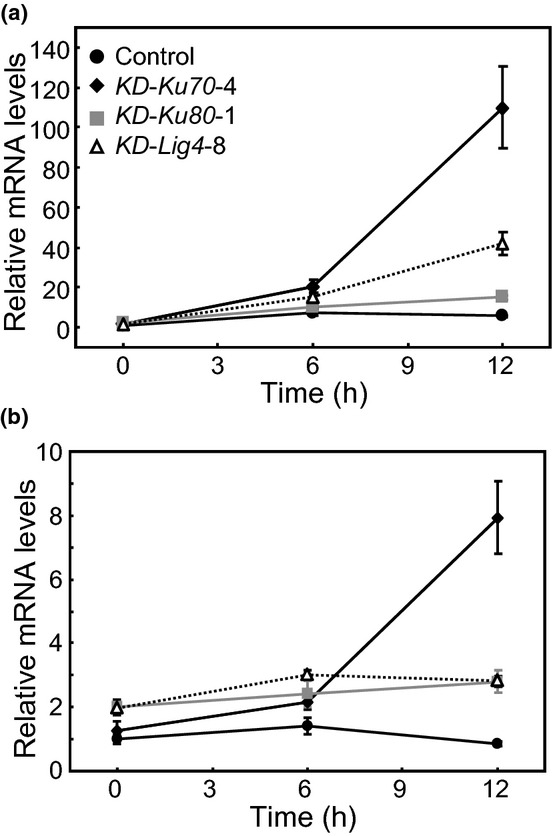
Effect of suppression of the nonhomologous end joining (NHEJ) pathway on the expression of genes involved in homologous recombination (HR). Seven-day-old seedlings of rice (*Oryza sativa*) wild-type, *KD-Ku70*, *KD-Ku80* and *KD-Lig4* seedlings were treated with water containing 5 μM bleomycin and incubated for 12 h in the growth chamber. Quantitative reverse transcriptase-polymerase chain reaction (RT-PCR) analysis of *OsRad51A2* (a) and *OsBRCA1* (b) transcripts in control, *KD-Ku70*, *KD-Ku80* and *KD-Lig4*. Relative transcript levels were normalized to *OsAct1* mRNA. Error bars represent ± SD of three individual experiments.

We further analyzed the transcriptional responses to bleomycin treatment of *OsKu70/80* and *OsLig4* suppression plants using an Agilent rice 44K microarray. The expression levels of HR-related genes are shown in Table 1. Transcript levels of not only *OsBRCA1* and *OsRad51A2*, but also *OsDMC1A* and *OsDMC1B*, the meiotic Rad51 paralog (Metkar *et al*., 2004), were induced in *KD-OsKu70* and *KD-OsLig4* rice plants relative to control plants under treatment with bleomycin (Table 1). In addition, under DNA-damaging conditions, the expression of *OsRad54*, one of the key proteins necessary for HR and DNA repair (Osakabe *et al*., 2006), and *OsTOP3* (putative topoisomerase 3) (Singh *et al*., 2010) was increased in *KD-OsKu70* plants. Putative *OsBARD1* – a BRCA1-associated RING domain protein (Singh *et al*., 2010) – was up-regulated in *KD-OsLig4* plants, but not in control plants, whereas no differences in the expression of HR-related genes other than *OsBRCA1* were noted between *KD-OsKu80* and control plants.

**Table 1:** Expression data of homologous recombination (HR)-related genes in control, *KD-Ku70*, *KD-Ku80* and *KD-Lig4* rice (*Oryza sativa*) plants with (12 h) or without (0 h) 5 μM bleomycin treatment

Accessions	Description	Control (12 h)		*KD-Ku70* (0 h)		*KD-Ku70* (12 h)		*KD-Ku80* (0 h)		*KD-Ku80* (12 h)		*KD-Lig4* (0 h)		*KD-Lig4* (12 h)	
Log_10_ ratio	*P*	Log_10_ ratio	*P*	Log_10_ ratio	*P*	Log_10_ ratio	*P*	Log_10_ ratio	*P*	Log_10_ ratio	*P*	Log_10_ ratio	*P*
Os05g0512000	OsBRCA1	−0.44	0.19	−0.02	0.92	**0.68**	**0.00**	0.01	0.93	**0.32**	**0.03**	**0.39**	**0.00**	**0.52**	**0.00**
Os01g0164900	OsBRCA2	0.00	1.00	0.00	1.00	0.41	0.21	−0.13	0.83	0.00	1.00	0.15	0.76	0.00	1.00
Os04g0635900	MRE11	−0.06	0.30	0.01	0.91	−0.10	0.12	0.00	0.98	−0.12	0.05	−0.04	0.50	−0.08	0.20
Os01g0948100	OsMUS81	−0.13	0.04	−0.15	0.02	−0.10	0.12	−0.14	0.03	−0.13	0.04	0.00	0.96	−0.04	0.56
Os02g0497500	Rad50	−0.02	0.72	0.03	0.66	0.09	0.17	−0.07	0.27	−0.06	0.36	−0.04	0.55	0.04	0.58
Os11g0615800	OsRad51A1	−0.12	0.07	0.11	0.08	0.26	0.00	0.25	0.00	0.17	0.01	−0.05	0.41	−0.20	0.00
Os12g0497300	OsRad51A2	**0.84**	**0.00**	0.00	1.00	**1.86**	**0.00**	−0.19	0.01	**0.57**	**0.00**	0.50	0.10	**1.45**	**0.00**
Os05g0121700	OsRad51B	−0.43	0.00	0.05	0.73	−0.01	0.90	−0.10	0.39	−0.23	0.09	−0.09	0.51	0.09	0.48
Os01g0578000	OsRad51C	−0.09	0.17	−0.04	0.51	−0.07	0.26	0.01	0.90	−0.02	0.70	−0.07	0.28	−0.09	0.17
Os09g0104200	OsRad51D	−0.02	0.72	−0.09	0.15	−0.07	0.25	0.03	0.67	−0.02	0.72	−0.02	0.79	−0.04	0.56
Os02g0762800	OsRad54	0.00	1.00	−0.26	0.65	**0.72**	**0.00**	0.00	1.00	0.05	0.93	−0.05	0.93	0.26	0.04
Os05g0392400	OsRad54-like	−0.17	0.01	0.02	0.77	−0.10	0.11	−0.16	0.02	−0.18	0.01	−0.03	0.63	0.01	0.85
Os10g0487300	OsNBS1	−0.16	0.01	−0.09	0.16	−0.21	0.00	0.01	0.81	−0.12	0.06	−0.06	0.36	−0.15	0.02
Os04g0433800	OsRecQl4	−0.36	0.00	0.04	0.59	0.20	0.00	0.20	0.00	0.01	0.94	**0.39**	**0.00**	0.16	0.02
Os01g0273000	DSS1/SEM1	0.05	0.46	0.04	0.55	0.06	0.33	0.20	0.00	0.15	0.02	0.14	0.02	0.11	0.09
Os02g0562100	XRCC3	−0.09	0.31	0.07	0.50	0.17	0.03	0.17	0.05	0.12	0.15	0.25	0.00	0.18	0.04
Os03g0165000	TOP3	−0.40	0.01	0.10	0.49	**0.38**	**0.00**	0.14	0.19	0.06	0.61	**0.36**	**0.00**	0.25	0.03
Os09g0500600	TOP3	−0.01	0.83	0.01	0.93	−0.04	0.56	−0.08	0.22	−0.06	0.38	−0.09	0.16	−0.09	0.21
Os05g0509700	SSB	−0.11	0.07	−0.08	0.21	0.02	0.78	0.07	0.23	0.02	0.75	0.15	0.02	0.06	0.37
Os01g0642900	SSB	−0.06	0.35	0.05	0.40	0.20	0.00	0.10	0.12	0.00	0.95	0.14	0.03	0.10	0.09
Os04g0648700	EME1	0.16	0.01	0.16	0.01	0.23	0.00	0.04	0.51	0.08	0.19	0.09	0.17	0.09	0.15
Os11g0146800	OsDMC1A	0.26	0.00	0.00	0.98	**0.72**	**0.00**	−0.13	0.04	0.20	0.00	−0.12	0.07	**0.66**	**0.00**
Os12g0143800	OsDMC1B	**0.50**	**0.00**	0.21	0.20	**1.32**	**0.00**	0.05	0.71	**0.40**	**0.00**	0.06	0.62	**1.64**	**0.00**
Os09g0280600	OsMND1	0.09	0.14	0.11	0.08	0.17	0.01	0.20	0.00	0.18	0.00	−0.03	0.64	−0.05	0.46
Os01g0901200	RecA	−0.17	0.01	−0.08	0.22	0.08	0.22	0.01	0.92	−0.06	0.35	−0.02	0.77	−0.07	0.26
Os03g0639700	RecA	−0.01	0.86	−0.02	0.80	−0.07	0.28	0.01	0.82	−0.03	0.63	0.00	0.97	0.01	0.83
Os11g0302700	RecA	−0.06	0.36	−0.10	0.14	−0.13	0.05	−0.07	0.26	−0.11	0.09	−0.19	0.00	−0.19	0.01
Os02g0710800	RecG	−0.12	0.06	−0.06	0.32	−0.12	0.06	−0.04	0.54	−0.13	0.04	−0.02	0.75	−0.05	0.41
Os05g0486600	BARD1	0.16	0.03	0.08	0.31	0.03	0.71	−0.02	0.77	−0.21	0.01	**0.32**	**0.00**	**0.39**	**0.00**
Os04g0512400	BARD1	−0.49	0.00	0.00	0.95	0.23	0.00	0.23	0.00	0.20	0.00	**0.47**	**0.00**	**0.37**	**0.00**

The log_10_ ratios of HR-related genes in control, *KD-Ku70*, *KD-Ku80* and *KD-Lig4* plants with (12 h) or without (0 h) 5 μM bleomycin treatment compared with those in control plants without bleomycin treatment are presented. Bold characters represent genes whose expression is significantly induced (log_10_ ratio > 0.3 and *P* < 0.01).

## Discussion

Here, we have demonstrated that the suppression of NHEJ-related gene expression causes decreased *Agrobacterium*-mediated stable transformation of the rice genome, especially in *KD-OsLig4* (Figs 2, 3). Lig4 proteins interact with XRCC4 and catalyze the ligation step of DSBs in the NHEJ pathway (Mladenov & Iliakis, 2011; Symington & Gautier, 2011). The most generally accepted mechanisms of T-DNA integration into the plant genome are the strand-invasion model and the DSB repair model depending on the NHEJ pathway. The results presented here suggest that NHEJ is the major pathway of T-DNA integration into the rice genome, at least when rice scutellum-derived calli are used for transformation, and that the OsLig4 protein may play an important role in this process.

The decreased stable transformation observed in *KD-OsKu70* and *KD-OsKu80* plants and *OsKu70*^*+*/−^ plants is in accordance with a previous report (Li *et al*., 2005), in which a decreased frequency of T-DNA integration was observed in *atku80* knockout mutants of Arabidopsis by root tumorigenesis assay. In addition, mRNA levels of *OsKu70* and *OsRad51A2*, which are well-known genes associated with DNA damage, are higher in calli than in leaves, roots and anthers (Yang *et al*., 2010). Yadav *et al*. (2009) have revealed an enrichment of DNA repair gene expression in Arabidopsis shoot apical meristem stem cells. Furthermore, consistent with previous observations (Hong *et al*., 2010), we found that knockout of the *OsKu70* gene resulted in a growth defect of calli (Fig.b), but had no effect on the growth of intact plants. Summarizing these results, DSBs arise constantly in rice callus as a result of active cell division. These DSBs are repaired mainly by the NHEJ pathway. DSBs are toxic for rice callus if not repaired correctly by the NHEJ pathway. T-DNA has been shown in several cases to be inserted into DSB sites (Salomon & Puchta, 1998). Thus, T-DNA is most likely to be integrated into the rice genome via NHEJ using endogenously induced DSBs.

There was no correlation between the transcript levels of *Ku80* and LUC luminescence derived from T-DNA stable transformation in *KD-OsKu80* rice calli (Figs 1b, 2d,e). It has been shown that the Ku70/80 protein functions as a heterodimer to repair DSBs, with the two constituents stabilizing each other (Smider *et al*., 1994). Accordingly, these results may be attributed to a decrease in protein levels of both OsKu80 and OsKu70 caused by the down-regulation of the expression of *OsKu80* mRNA in *KD-Ku80* rice calli.

We also detected decreased *Agrobacterium*-mediated stable transformation in *KD-OsLig4* rice callus. However, it has also been reported that the absence of AtLig4 does not affect the frequency of T-DNA integration in an *in vitro* root tumorigenesis assay (van Attikum *et al*., 2003). Although we cannot rule out the possibility that AtLig4 and OsLig4 act differently in each species in the process of *Agrobacterium*-mediated stable transformation, differences in the assay system itself could lead to different results. In the root tumorigenesis assay, the efficiency of T-DNA integration was evaluated by tumor formation in roots that were grown via cell division and cell expansion for 4–5 wk after *Agrobacterium* infection; many environmental factors are involved in this process. Our *Agrobacterium*-mediated stable transformation assay system allows transient and stable expression to be visualized using a sensitive reporter gene, and thus stable transformation events can be detected rapidly (within 7–10 d after *Agrobacterium* infection).

It has been shown recently in Arabidopsis that the epidermal cells of the root tip are enlarged and show increased nuclear DNA content in response to DSBs derived from treatment with the genotoxic agent zeocin, indicating that DSBs induce the onset of the endocycle (Adachi *et al*., 2011). In other words, DNA-damaged cells in Arabidopsis could select the repair of DNA damage, the induction of cell death or, alternatively, the induction of the endocycle to avoid cell death. We speculated that a fraction of cells with integrated T-DNA could enter the endocycle as a result of DSB signals, as DSBs have been reported to be major integration sites of T-DNA (Salomon & Puchta, 1998). In contrast with Arabidopsis, the endocycle has never been reported in rice except in the endosperm. We have recently confirmed that this is true, even after genotoxic stress treatment inducing DSBs (Endo *et al*., 2012). According to this concept, the frequency of T-DNA integration could be underestimated in Arabidopsis, as *AtKu70/80* or *AtLig4* mutants accumulate DSBs as a result of insufficient NHEJ repair.

Decreased T-DNA integration has also been reported in *atku80* and *atlig4* mutants when analyzed using an *in planta* floral dip transformation assay (Friesner & Britt, 2003), whereas no differences were detected between *atku80* mutants and wild-type plants (Gallego *et al*., 2003). Furthermore, Ziemienowicz *et al*. (2008) have reported that Arabidopsis type I DNA ligase (Lig1) can function as a ligase for T-DNA *in vitro*, suggesting that T-DNA integration may be catalyzed by Lig1 via the strand-invasion model in the case of *in planta* floral dip transformation. The presence of a micro-homology sequence in the T-DNA integration site identified via *in planta* floral dip transformation (Brunaud *et al*., 2002) also supports this idea. Micro-homology-dependent integration of T-DNA into the rice genome was also reported when scutellum-derived calli were used for transformation (Sha *et al*., 2004). To better understand the mechanism of T-DNA integration in plants, the effect of the suppression of Arabidopsis or rice Lig1 on the efficiency of T-DNA integration via *in planta* floral dip or callus transformation should be investigated.

We observed a sterile phenotype and delayed callus proliferation in *OsKu70* mutants. By contrast, Arabidopsis *ku70*, *ku80* and *lig4* mutant plants are viable and exhibit a fertile phenotype (Bundock *et al*., 2002; van Attikum *et al*., 2003; Friesner & Britt, 2003). In addition, no significant difference in callus formation and callus regeneration from roots was observed between Arabidopsis *ku80* mutant and wild-type plants (Li *et al*., 2005). These differences suggest that the defect in the NHEJ pathway that depends on *Ku70*, *Ku80* and *Lig4* is more severely affected in rice than in Arabidopsis. Furthermore, it has been reported that cells with DNA damage enter into the endocycle to prevent their proliferation and cell death in Arabidopsis (Adachi *et al*., 2011). Consequently, mutations of Arabidopsis *ku70*, *ku80* and *lig4* may have little direct effect on viability and productivity.

Of all rice tissues, HR events occur with highest frequency in callus tissue under normal conditions (Yang *et al*., 2010). Consistent with this observation, we detected LUC luminescence derived from reconstituted recombination substrate in both control and transgenic calli; HR activity in *KD-Ku70* and *KD-Lig4* transgenic calli was two- to three-fold higher than in control calli (Fig.d,e). Thus, the frequency of HR seemed to be enhanced by suppression of the NHEJ pathway in rice plants, whereas there was no difference in the frequency of DSB-inducible HR between control and transgenic calli (Fig. S1b). In this study, to eliminate the effect of the efficiency of T-DNA integration on I-*Sce*I expression levels, we analyzed LUC luminescence by monitoring DSB-inducible HR at 5 d after infection with *Agrobacterium* harboring an I-*Sce*I expression vector. It was assumed that LUC luminescence derived from DSB-inducible HR might not be detected correctly because I-*Sce*I expression levels derived from free T-DNA may be very low. Alternatively, it is possible that no differences were observed in the frequency of DSB-inducible HR between control and transgenic calli because DSBs induced by I-*Sce*I expression at the two I-SceI sites flanking the *codA* expression cassette of LU-UC recombination substrates were repaired via the single-strand annealing (SSA) pathway. It has been reported that DSBs flanking directly repeated sequences are repaired mainly by the SSA pathway using homologous sequences (Siebert & Puchta, 2002). In future, evaluation of the frequency of DSB-inducible HR should be performed with *KD-Ku70/80* or *KD-Lig4* harboring a recombination substrate and a stably integrated I-*Sce*I expression cassette driven by a chemically inducible system. To assess HR frequency in *KD-Ku70/80* or *KD-Lig4* in detail, the establishment of an HR assay system in rice is also required to detect the reconstruction of the recombination substrate only via HR, for example, intrachromosomal HR reporter with indirect repeats (Gherbi *et al*., 2001) or interchromosomal HR reporter (Molinier *et al*., 2004).

A large number of genes, including HR-related genes, are up-regulated in response to ionizing radiation (Nagata *et al*., 2005; Culligan *et al*., 2006; Ricaud *et al*., 2007). In addition, previous studies of Arabidopsis *ku80* mutants have shown that several genes, including DSB repair genes, display transcriptional induction (West *et al*., 2004). Consistent with these findings, we demonstrate here that the transcript levels of *OsRad51A2* and *OsBRCA1* are higher in *KD-Ku70*, *KD-Ku80* and *KD-Lig4* plants than in control plants under conditions of DNA damage (Fig.). Considering the transcriptional induction of HR-related genes in *KD-Ku70*, *KD-Ku80* and *KD-Lig4*, suppression of the NHEJ pathway causes the accumulation of DNA damage under stressful conditions, such as active cell division and treatment with DNA-damaging agents, and the HR pathway is then activated and DNA damage repaired as a result of the induction of HR-related gene expression. Recently, it has been shown that Ku proteins interact with a number of proteins and function not only in the NHEJ pathway for DSB repair, but also in transcriptional regulation and signal transduction in mammals (Bertinato *et al*., 2003; Brenkman *et al*., 2010; Zhang *et al*., 2011; Fell & Schild-Poulter, 2012). Wang *et al*. (2010) have identified Arabidopsis ovate family protein 1 (AtOFP1) as a novel AtKu70-interacting protein using yeast two-hybrid screening and pull-down assay. In this report, suppression of *OsKu70* expression has a major effect on HR-related genes, such as *OsRad51A2* and *BRCA1* (Fig., Table 1), therefore suggesting that *OsKu70* may also be involved in the signaling pathway of the DSB response.

In conclusion, our results clearly show the involvement of the NHEJ pathway in the *Agrobacterium*-mediated stable transformation in rice and the presence of the competitive and complementary relationship between the NHEJ and HR pathways for DSB repair in rice. Although it takes a relatively long time to set up an assay system in rice relative to Arabidopsis because of its longer life-cycle, a well-established evaluation system in rice could be applied to other important crops in Gramineae.

In contrast with random T-DNA integration, sequence-specific integration of T-DNA into the endogenous homologous sequence – gene targeting (GT) – occurs through the HR pathway (Smithies *et al*., 1985; Paszkowski *et al*., 1988; Müller, 1999; Iida & Terada, 2004). As a result, it has been reported that GT events of endogenous genes by HR occur on the order of 0.01–0.1% compared with random integration in higher plants (Paszkowski *et al*., 1988; Offringa *et al*., 1990; Puchta *et al*., 1996). Our data indicate that suppression of the NHEJ pathway is expected to cause an increase in the occurrence of sequence-specific integration by HR and inhibition of random integration by NHEJ, resulting in a synergistic effect that will improve GT experiments. In fact, it has been reported that deletion of the *Ku* or *Lig4* gene can increase the frequency of GT in several organisms, such as fungi (de Boer *et al*., 2010; Ushimaru *et al*., 2010; Nakazawa *et al*., 2011), bacteria (Zhang *et al*., 2012) , birds (Iiizumi *et al*., 2008), mammals (Bertolini *et al*., 2009; Fattah *et al*., 2008; Iiizumi *et al*., 2008) and plants (Tanaka *et al*., 2010; Jia *et al*., 2012). We are progressing towards the establishment of a high-efficiency GT procedure using KD rice plants targeting NHEJ-related genes.

## Notes

Fig. S1 Luciferase (LUC) luminescence of DNA double-strand break (DSB)-inducible homologous recombination (HR) events by transient I-*Sce*I expression in control, *KD-OsKu70* and *KD-OsLig4* rice calli.
