# Supplementary material for: Suppression of Ku70/80 or Lig4 leads to decreased stable transformation and enhanced homologous recombination in rice
Source: New Phytol. 2012 Oct 10;196(4):1048–59. doi: 10.1111/j.1469-8137.2012.04350.x (PMC3532656; doi:10.1111/j.1469-8137.2012.04350.x)
Supplement: Fig. S1 — Luciferase (LUC) luminescence of DNA double-strand break (DSB)-inducible homologous recombination (HR) events by transient I-SceI expression in control, KD-OsKu70 and KD-OsLig4 rice calli. [file nph0196-1048-SD1.ppt]

## Slide 1
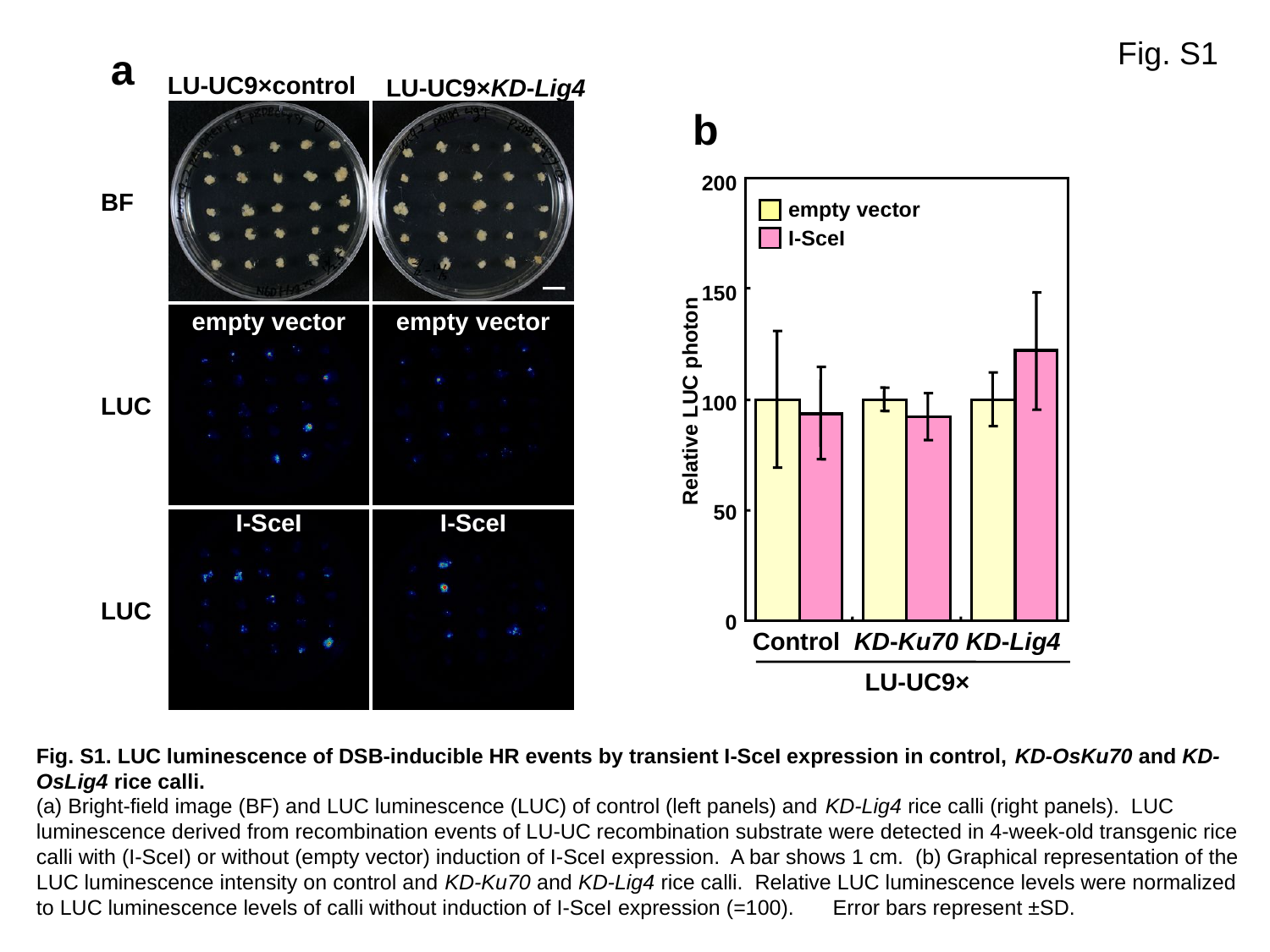

Fig. S1
a
LU-UC9×control
LU-UC9×KD-Lig4
BF
empty vector
empty vector
LUC
I-SceI
I-SceI
LUC
b
200
empty vector
I-SceI
150
Relative LUC photon
100
50
0
KD-Ku70
KD-Lig4
Control
LU-UC9×
Fig. S1. LUC luminescence of DSB-inducible HR events by transient I-SceI expression in control, KD-OsKu70 and KD-OsLig4 rice calli.
(a) Bright-field image (BF) and LUC luminescence (LUC) of control (left panels) and KD-Lig4 rice calli (right panels). LUC luminescence derived from recombination events of LU-UC recombination substrate were detected in 4-week-old transgenic rice calli with (I-SceI) or without (empty vector) induction of I-SceI expression. A bar shows 1 cm. (b) Graphical representation of the LUC luminescence intensity on control and KD-Ku70 and KD-Lig4 rice calli. Relative LUC luminescence levels were normalized to LUC luminescence levels of calli without induction of I-SceI expression (=100). 　Error bars represent ±SD.
